# Cognitive asymmetry in rats in response to emergent vs. disappearing affordances

**DOI:** 10.1007/s10071-024-01886-2

**Published:** 2024-07-15

**Authors:** Wojciech Pisula, Klaudia Modlinska, Anna Chrzanowska, Katarzyna Goncikowska

**Affiliations:** Institute of Psychology, Polish Academy of Sciences, Jaracza 1, Warsaw, 00-378 Poland

**Keywords:** Animal cognition, Affordances, Cognitive asymmetry, Rat, Exploratory behavior, Neotic preferences

## Abstract

This study examines the effects of novel environmental changes on the behavior of rats in an experimental chamber. We hypothesized that newly discovered opportunities, detected by the animal’s cognitive system, would motivate greater investigation of environmental changes than comparable changes that prevent a given behavior. Three experiments differed in the emergence vs. elimination of affordances represented by open or closed tunnels. In Experiment 1, rats were habituated to a chamber with all four tunnels closed, and then two tunnels were opened. In Experiment 2, rats were habituated to a chamber where all four tunnels were open, and then two tunnels were closed. In Experiment 3, rats were habituated to a chamber with two open tunnels on one side, and two closed tunnels on the other. Then, the arrangement of open and closed tunnels was swapped. Results of the Exp. 1 show that the rats responded by spending more time near the newly opened tunnels and less time near the closed tunnels, the central zone, and the transporter. This suggests that rats are more motivated to investigate the environmental change combined with the emergent affordance (opening of the tunnels) than the environmental change alone. In Exp. 2, the rats responded by spending more time near the open tunnels and less time in the central zone. This suggests that the rats are more triggered by the available affordances (open tunnels) than by the environmental change (closed tunnels). Finally, in Exp. 3, the rats responded by spending more time near the newly opened tunnels and less near the central zone. However, they did not spend less time near the newly closed tunnels. These results suggest that rats process both the novelty itself and the emergence/disappearance of available affordances. The results are discussed regarding the cognitive asymmetry in the perception of emergent vs. disappearing affordances. It is proposed that the rat’s cognitive system is specialized for detecting newly emergent environmental opportunities/affordances rather than novelty in general.

## Introduction

The field of animal research in psychology has evolved significantly, transitioning from the conventional “animal model” method to a modern framework that emphasizes processes rather than particular species (Maestripieri 2005). Vonk (2021, p. 156) effectively encapsulates this change by suggesting that comparative psychologists should explore the development, function, and mechanisms of behavior across various species within a biological context. In alignment with this approach, our research program investigates how organisms adapt to environmental changes. These changes, which individuals perceive as new or unfamiliar experiences, are essential for them to adapt to new situations. This process occurs at multiple levels, including cognitive and protocognitive processes, which involve capacities for controlling individual behavior by means of adaptive response to environmental information (Godfrey-Smith 2002; Pisula 2020).

One specific way in which organisms respond to novelty is through exploration. Exploration involves actively gathering information about the environment in ways that allow individuals to prepare for future challenges, such as avoiding predators, adverse weather conditions, or securing access to food (Berlyne 1963). Exploration encompasses a range of behavioral phenomena, including responses to novelty, risk assessment, arousal, locomotor activity, habituation, and memory. Even when a novel stimulus does not offer intrinsic rewards or biological benefits, it can act as a positive motivator due to its strong motivational properties (Costa et al. 2014). The inherent interest in novelty displayed by organisms, a central aspect of exploratory behavior, is considered an evolutionary prerequisite for complex learning and helps organisms acquire adaptive behavioral repertoires (Farahbakhsh and Siciliano 2021).

Investigating responses to new objects is a central field of study in animal behavior and cognitive psychology. This research provides valuable insights into the mechanisms of learning, memory, and curiosity. Early work by Berlyne in the 1950s established the importance of novelty and curiosity as fundamental drivers of exploratory behavior, laying the groundwork for extensive future investigations. Berlyne’s pioneering studies have since been expanded to include a variety of species, such as rats (Berlyne 1955), domestic rabbits (Andersson et al. 2014), and even octopuses (Vergara-Ovalle et al. 2023). These studies underscore the universal significance of exploring novel objects in understanding animal cognition.

In particular, novel object recognition (NOR) in rats has become a widely used paradigm to assess memory. It has significantly contributed to our understanding of the neurobiological basis of cognition (Antunes and Biala 2012). For instance, Arqué et al. (2008) demonstrated the role of dual-specificity tyrosine-regulated kinase-1 A in spatial learning strategies and NOR. Their research provided clear evidence that removing a single gene can profoundly affect phenotype, suggesting that the haploinsufficiency of a single gene might impair cognitive functions and stress-coping mechanisms.

The importance of NOR extends beyond mammals. Recent studies have begun to explore novel object recognition in other species, such as *Octopus maya*, revealing complex cognitive abilities in invertebrates (Vergara-Ovalle et al. 2023). These findings broaden our understanding of animal cognition and challenge the traditional mammalian-centric view of cognitive research. They highlight that approaches to novel objects vary significantly across species and even within species, reflecting underlying personality dimensions.

Studies of novel-object-related behavior in various species provide invaluable insights into cognitive and behavioral adaptations to environmental challenges. From Berlyne’s seminal work to contemporary research across a wide range of species, this body of literature underscores the complexity of exploratory behavior and its underlying mechanisms. As research continues to evolve, it will undoubtedly deepen our understanding of the cognitive worlds of animals, revealing the multifaceted nature of curiosity and exploration. For example, Andersson et al. (2014) found that novel-object and anti-predator behaviors in domestic rabbits are influenced by different personality dimensions, suggesting a complex interplay between personality and cognitive exploration. Similarly, Blaszczyk (2017) found that boldness toward novel objects predicted predator-inspection behavior in wild vervet monkeys, illustrating the adaptive importance of novelty exploration.

Despite extensive research, challenges in interpreting NOR test results have led to critical assessments of the paradigm. Takola et al. 2021 conducted a meta-analysis of the novel-object paradigm, emphasizing the need for a nuanced understanding of novelty responses and their implications for animal cognition studies. This reflects a broader trend in cognitive and behavioral research, highlighting the importance of methodological rigor and contextual interpretation of findings. The ongoing refinement of NOR methodologies and their application across diverse species will continue to enhance our comprehension of the intricate relationships between novelty, cognition, and behavior in the animal kingdom.

Due to the arguments outlined above, one may also postulate that the stimulus-object applied in the studies should fulfill some requirements, namely ecological validity. In this context, the stimulus-object should invite a particular behavioral activity of the individual, as Stoffregen (2003) described. In our experimental setup, we utilize wooden tunnels to encourage rats to explore and climb on top of them. Throughout our studies on exploration, we have developed a protocol and specialized testing arena (Pisula and Modlinska 2020) designed to study free exploration in small laboratory mammals such as rats, mice, and short-tailed opossums (e.g., Pisula et al. 2012; Modlinska et al. 2019; Chrzanowska et al. 2022). To ensure the highest ecological validity of our analyses, we prioritize minimizing animal stress during the experiment. We effectively achieve this by introducing a prolonged habituation period before introducing low-intensity novelty. The previous studies (Modlinska et al. 2019, 2020; Pisula et al. 2019, 2021, 2022; Chrzanowska et al. 2022) have provided initial insights into the phenomena we are investigating. Summarizing this phase of research, Pisula and Modlinska (2023 p. 297) note that the cognitive system of rats appears to process increased environmental complexity intensely and effectively. On the other hand, reduced environmental complexity elicits less exploratory behavior and requires less cognitive effort. However, the adaptive significance of both environmental changes (increase vs. decrease in complexity) seems comparable since knowledge of the disappearing opportunity is as important as knowledge of the emerging opportunity. The higher cognitive sensitivity of rats to emerging opportunities compared to their low sensitivity to disappearing opportunities is an interesting cognitive phenomenon that reveals new elements of the structure of the animal mind and deserves more attention. The emergence of new possibilities for behavioral outcomes with respect to new environmental properties is often referred to as affordances (Gibson 1977).

Affordances are properties of an animal’s environment that influence its behavior and provide opportunities for action (Greeno 1994; Turvey 1992; Stoffregen 2003). Our studies have highlighted the concept of “perceived affordance” by the animal’s cognitive system. It is important to note that the affordance itself is not an independent variable; the animal must recognize and process its qualitative and quantitative properties. Using the concept of affordance to elicit behavior may provide better insights into the results of our previous studies (Modlinska et al. 2019, 2020; Pisula et al. 2019, 2021, 2022; Chrzanowska et al. 2022). In the present study, we operationalize this concept through the state of the tunnels (open vs. closed). The initiation of the possibility to engage in a new activity (i.e., entering the tunnel) is a critical factor that triggers a series of actions typically described as expressions of curiosity. The purpose of this study is to explore this phenomenon further.

In this paper, we present three experiments on novelty-related behavior. The emergent affordances are represented at the operational level by the emergent tunnel entrances, allowing the animals to explore new sections of the test arena. Elimination of the affordances is achieved by closing the tunnel entrances. Environmental complexity is maintained at a stable level throughout the experiment. With the precise control of the animal’s experience prior to the start of the experiment, we should be able to separate the effect of the experimental manipulation on the animal’s response to novelty from other confounding factors, such as environmental complexity. Thus, animals will be exposed to environmental changes through the emergence vs. elimination of affordances represented by open vs. closed tunnels. Our current research method may appear to be a combination of our research strategy to date (Modlinska and Pisula 2020) and the inspiring study by Alvernhe et al. (2012). However, their study focused mainly on the rat’s ability to construct or update cognitive maps, whereas our study explores such aspects as activity motivation and time and effort budgeting. Our perspective adopts the concept of affordance as a cognitive phenomenon that plays the organizing role for actual conduct. The environmental change may open or close a new possibility for animal actions, which the animal’s cognitive system should detect. Research has shown that animals perceive affordances in a way that exhibits action scaling, meaning that they make choices about when to transition from one behavior to another based on the affordances available in the environment (Wagman et al. 2017). For example, in a study with dogs, researchers investigated the heights at which dogs chose to transition from reaching with the head only to rearing (i.e., reaching with the head plus torso). They found that this transition occurred at a taller height for tall dogs than for short dogs but at the same ratio of shoulder-height-to-treat-height for both groups (Wagman et al. 2017). These results suggest that the perception of affordances is supported by the detection of a stable structured pattern of the stimulus field and available motor patterns that may be invariant across species.

If the novel event opens new possibilities, motivational factors should drive the individual to explore these opportunities and incorporate them into the cognitive map of the surroundings (see Pisula and Modlinska 2023, page 297, conclusion section, point 1). On this basis, we hypothesize that newly discovered opportunities, detected by the animal’s cognitive system, will motivate individuals to investigate the environmental change to a greater extent than a comparable environmental change which leads to the prevention of a given behavior activity.

On the operational level, the study aims to test the hypothesis that rats will exhibit a pronounced response to newly opened tunnels (emergent affordance) and a lower level of exploratory activity to newly closed tunnels (disappearing affordance) when the complexity and attractiveness of all test arena zones are maintained at a similar level. To achieve maximum control over the experimental variable, the study will consist of three formally independent experiments, further described as experiments #1, #2, and #3.

## Method

The general methodology, apparatus, and measurement techniques employed in this study were comparable to those utilized in our previous research (Modlinska et al. 2019, 2020; Pisula et al. 2019, 2021, 2022; Pisula and Modlinska 2020; Chrzanowska et al. 2022). In contrast to standard two-dimensional experimental setups, our approach provides a complex three-dimensional environment. Our protocol examines the exploration of a novel environment, rates of habituation, and responses to innocuous and low-intensity novelty within a familiar context in small mammals. The experimental chamber allows animals to explore different zones both horizontally and vertically, allowing them to navigate the test arena and find refuge in a safe space (e.g., a transporter). The animals are allowed to freely explore the designated experimental arena, which includes wooden tunnels as objects (also newly introduced objects) for the animals to explore. The tunnels allow animals to enter and hide. Novelty is introduced only after the animals have become familiar with the experimental setup. Depending on the specific experimental configuration, changes may be made in the number of tunnels or their characteristics.

### Apparatus

**Fig. 1:**
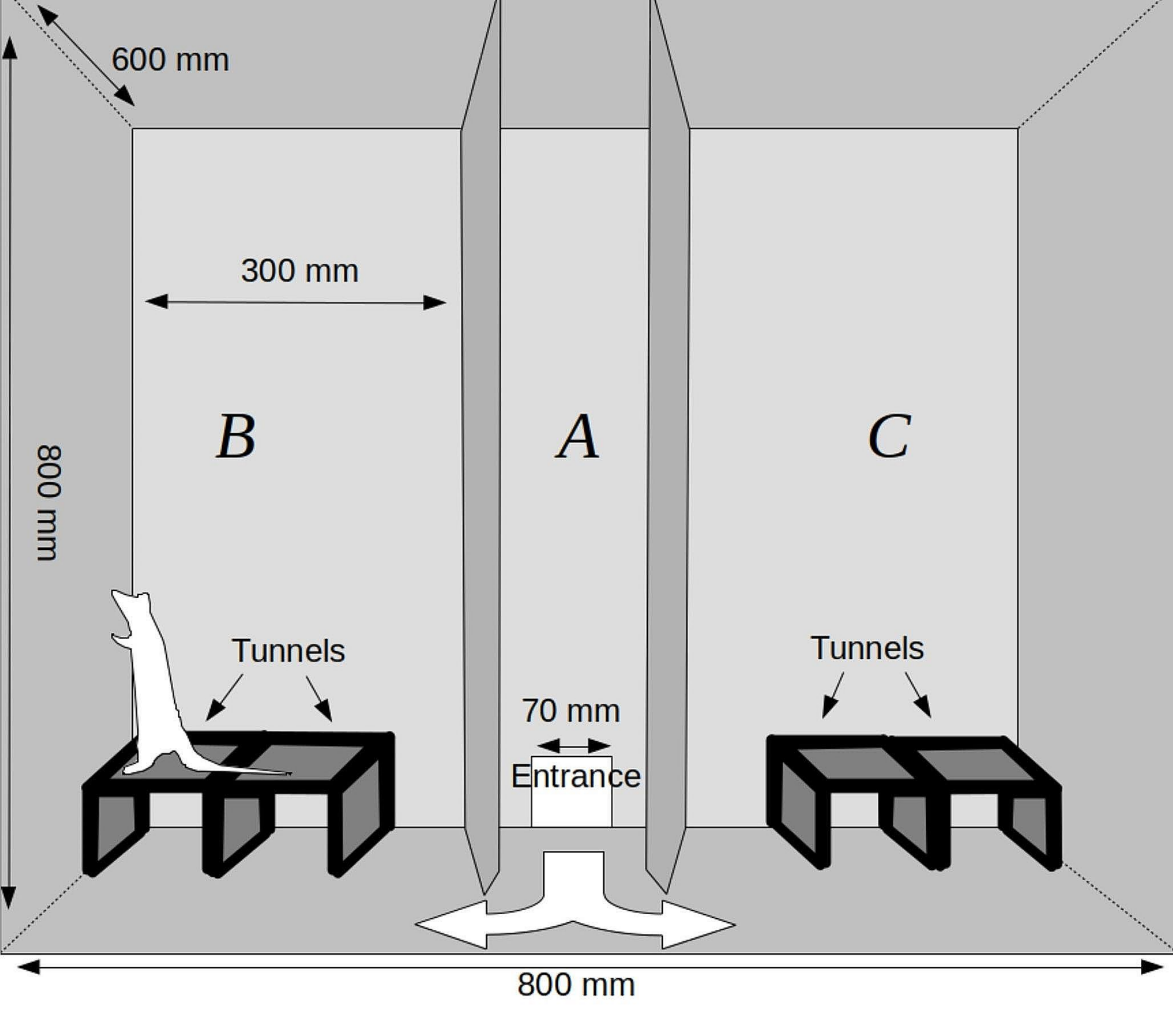
Schematic representation of our experimental box - frontal view through a plexiglass wall — more detailed description in the text. Please consult Pisula and Modlinska (2020) for all technical and procedural details

The test arena is a box measuring 800 mm in width, 800 mm in height, and 600 mm in depth (Fig. 1). The chamber is divided into three zones: A, B, and C. The front of the chamber is constructed of clear Plexiglas that can be raised to provide access to the test arena. The wooden division walls between the zones have triangular entrances (120 mm x 140 mm) at the bottom, which enables the animals to move freely between the chamber parts. Dividers are placed at an angle so as to prevent blind spots in the video recording from the top and from the front. The entrance to the chamber is placed in the central zone (A). The remaining zones, B and C, are where the objects allowing experimental manipulations are placed. The entire chamber is covered with a layer of washable varnish. Wooden tunnels are covered with washable paint. Tunnels provide a complex three-dimensional environment. In our experiments, we used tunnels (200 mm x 120 mm x 80 mm) made of hardwood and covered with washable dark brown paint. The tunnels are placed in zones B and C. The configuration of the tunnels is changed in one of the chamber zones before the first test trial, and this change introduces novelty to the experimental plan. Tunnel configuration is changed by manually moving them or adding/subtracting tunnels in two zones of the chambers. To introduce novelty in our present experiments, we modified the characteristics of the tunnels by either closing or opening them.

### Animals

As experimental subjects, we used 50 experimentally naïve male Lister Hooded rats (Modlinska & Pisula 2020). The subjects were assigned to three experiments (experiment #1–17 rats; experiment #2–16 rats; experiment #3–17 rats). The rats were housed in the vivarium of the Institute of Psychology, Polish Academy of Sciences, Warsaw, Poland. At the beginning of the study, the rats were approximately 60 days old and weighed approximately 250 g.

The rats were housed in groups of 3–4 in Tecniplast© Eurostandard type IV cages 610 mm × 435 mm × 215 mm with dust-free softwood granules Tierwohl Super© as bedding and ad libitum access to water and standard laboratory chow Labofeed H, WP Morawski, Kcynia, Poland. The day/night cycle was set at 12/12 h, with lights on at 8:00 a.m., temperature maintained at a constant 21–23 °C, and humidity at 45–60%.

### Procedure

The exploration test was designed to assess exploration behavior in response to the introduction of low-intensity novelty in a familiar context. All three experiments followed a similar protocol. They consisted of 10 sessions, with 7 familiarization sessions and 3 test sessions performed for each animal. Each session lasted 7 min and was carried out once a day for each animal. The trials were conducted during the light phase of the day/night cycle. It started around 10 am and lasted about 3 h. During the test, both the experimental room and the experimental area were kept in complete darkness.

**Fig. 2:**
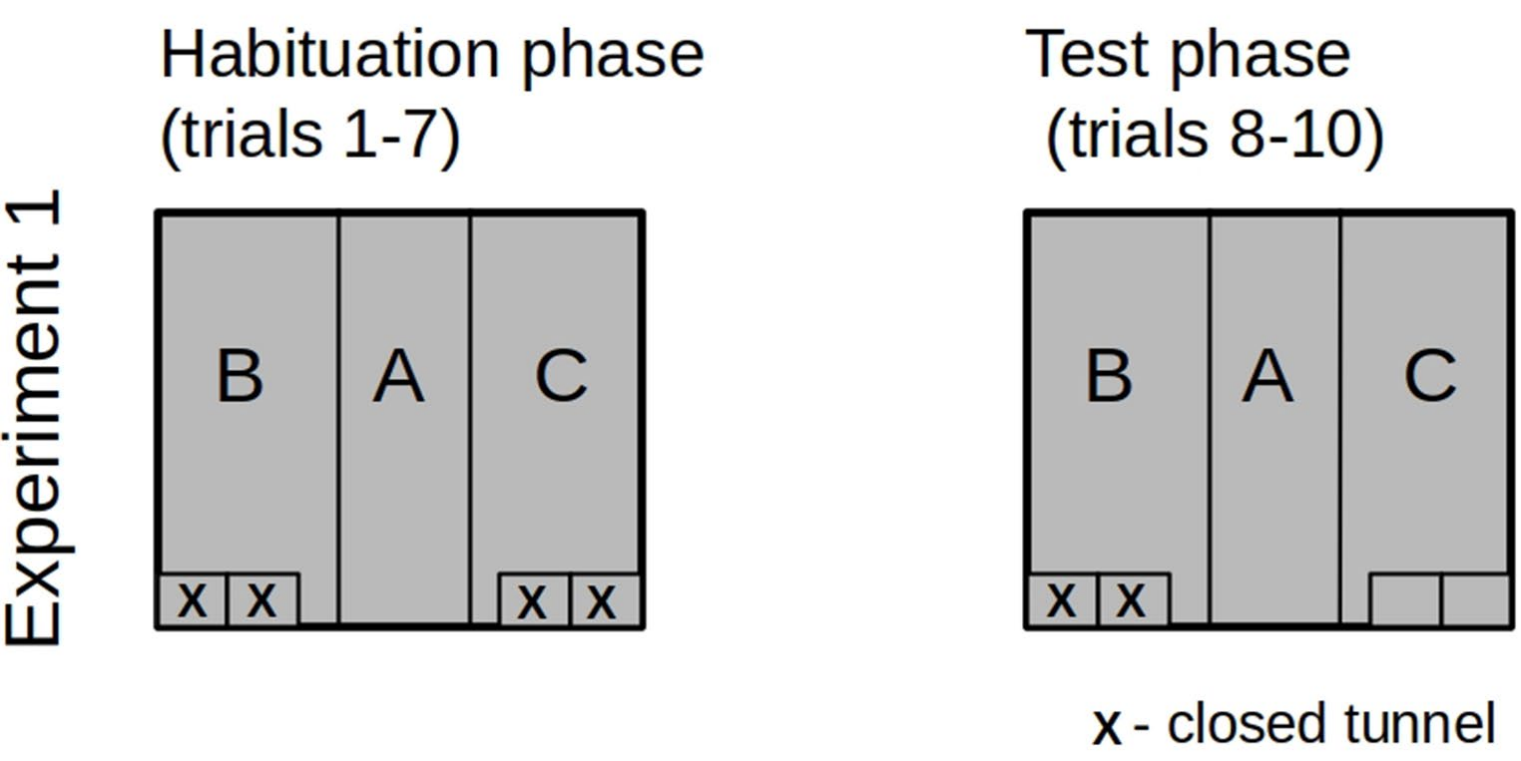
The tunnels’ configuration in the habituation and test phases in Experiment #1. “X” indicates a closed tunnel entrance

At the beginning of each session, a rat was removed from its home cage and placed in a small cylindrical cage called the “transporter”. The transporter had a diameter of 60 mm and doors that were 120 mm high and 100 mm wide. The transporter, with the subject inside, was then moved into the test room and positioned near the entrance to Zone A (Fig. 1). The entrance door was raised and left open for the duration of the experiment. The animal could either remain in the transporter or exit to freely explore the chamber. The tunnel configuration remained consistent throughout the habituation sessions. Each experimental group was habituated to different tunnel configurations, as shown in Figs. 2 and 4, and 6. The introduction of the novelty (i.e., the closing or opening of some tunnels) occurred before trial 8. The three subsequent trials were conducted with a new arrangement of tunnels.

After each session, the experimental arena, including the tunnels and the transporter, was meticulously cleaned with Virkon S (Bayer) to eliminate any odor cues left by the previous animal.

Each session was recorded using a video camera positioned approximately 1.5 m from the transparent front wall of the experimental chamber. The camera was set to night recording mode to allow for filming without daylight.

The three experiments described below differed in how we arranged the chamber layout during the familiarization and test trials.

### Data processing and statistical analyses

To encode the behaviors based on the recorded material, we used BORIS software (Friard & Gamba 2016), which allowed us to define selected behaviors and assess their duration and frequency. We scored the behaviors performed by the animals throughout the experimental trial. This allowed us to assign specific scores to the duration of each behavior, its frequency, and the total time an animal spent engaging in a given behavior. The following variables were measured: (1) time spent in the transporter (excluding latency to leave the transporter); (2) time spent in the central zone; (3) time spent in the unmodified zone of the chamber; (4) time spent in the modified zone of the chamber; (5) frequency of movement between zones (left/right/transporter) of the chamber; (6) time spent in contact with the tunnels in the unmodified zone of the chamber; and (7) time spent in contact with the tunnels in the modified zone of the chamber. Contact with the tunnels was defined as direct exploration, including sniffing, touching, vibrissae inspection, climbing, or touching with the body or tail.

The next step was to establish a reference value for the study samples. This was done by aggregating the last three familiarization trials (H5 to H7) and calculating the mean across all subjects/variables. The reference value was then referred to as trial H values, while the three test trials were referred to as T1, T2, and T3, respectively. We have chosen not to present the results of the first three Habituation trials as they serve only as a habituation phase and not as an element of a comparative analysis of the animals’ response to novelty. These data were then used in a repeated measures ANOVA analysis with H, T1, T2, and T3 as the within-subject factor Trial. The Greenhouse-Geisser sphericity correction was used whenever the sphericity assumption was violated. The *a priori* Helmert contrast tests followed the RM ANOVA, when a main effect was found significant. Differences were considered significant if *p* ≤ 0.05.

### Experiment #1

In Experiment 1, rats were habituated to the symmetrical arrangement of the chamber, with all four tunnels remaining closed. Before the test trials, two tunnels were opened (Fig. 2). Therefore, the novelty was introduced by opening the entrances to tunnels on one side of the experimental box. To avoid the effect of lateralization or visual-auditory cues, the novelty was implemented in the right zone (as described above - zone C) for half of the test rats and in the left zone (zone B) for the remaining half (mirror image of the experimental settings).

## Results of experiment #1

### Time budget allocation across the chamber zones

#### Time spent in the transporter

**Fig. 3:**
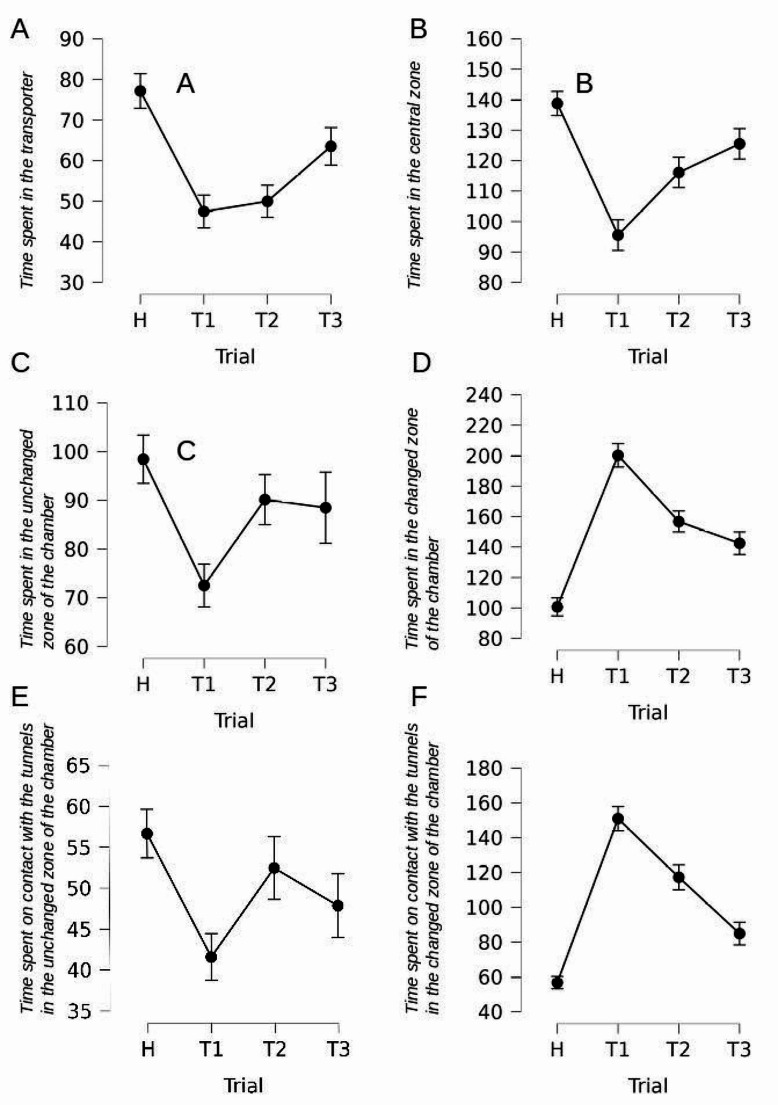
Statistically significant results of experiment #1. Time in seconds. Error bars show standard error

Rats shortened the duration of staying in the transporter [F(3,48) = 10.486, *p* < 0.001, η²*p* = 0.396]. Planned contrasts revealed that the effect showed up in all test trials (T1, T2, and T3) compared to trial H [t = 4.810, *p* < 0.001]. Additionally, there was an increase in time spent in transporter in trial T3 compared to trial T2 [t=-2.262, *p* = 0.028] - Fig. 3-A.

### Time spent in the central zone

Rats changed the time spent in the central zone of the chamber across the trials [F(3,48) = 14.624, *p* < 0.001, η²*p* = 0.478]. First, according to the planned contrasts, rats shortened the duration of staying in the central zone in trials T1, T2, and T3, as compared to trial H [t = 4.804, *p* < 0.001]. However, recovery of the time spent in the central zone was observed in trials T2 and T3 as compared to trial T1 [t=-4.341, *p* < 0.001] - Fig. 3-B.

### Time spent in the unchanged zone of the chamber

Rats responded by shortening the duration of staying in the unmodified (tunnels closed) zone of the chamber [F(3,48) = 3.802, *p* = 0.016, η²*p* = 0.192] in trial T1, T2, and T3 as compared to trial H [t = 2.294, *p* = 0.026]. Yet, the time spent in this zone increased in T2 and T3 compared to T1 [t=-2.469, *p* = 0.17] - Fig. 3-C.

### Time spent in the changed zone of the chamber

Rats prolonged the time spent in the changed (by opening the tunnels) zone of the chamber across the trials [F(3,48) = 34.360, *p* < 0.001, η²*p* = 0.682]. Planned contrasts revealed that this was observed in all test trials T1, T2, and T3 as compared to trial H [t=-8.136, *p* < 0.001]. There was also a gradual process of shortening the duration of staying in the modified zone of the chamber in trial T2 and T3 as compared to trial T1 [t = 5.900, *p* < 0.001], Fig. 3-D.

### Interactions with the open vs closed tunnels

#### Time spent on contact with the tunnels in the unchanged zone of the chamber

Rats responded with a shortening of the duration of interacting with tunnels in the unmodified (tunnels remained closed) zone of the chamber [F(3,48) = 3.555, *p* = 0.021, η²*p* = 0.182]. Planned contrasts revealed that the effect was observed in trials T1, T2, and T3 compared to trial H [t = 2.365, *p* = 0.022]. However, there was later an increase in the time spent on contact with tunnels in this zone in trials T2 and T3 compared to T1 [t=-2.042, *p* = 0.047], Fig. 3-E.

### Time spent on contact with the tunnels in the changed zone of the chamber

Rats immensely changed the duration of interaction with tunnels in the modified (by opening the tunnels) zone of the chamber [Greenhouse-Geisser correction, F(2.286, 36.570) = 42.926, *p* < 0.001, η²*p* = 0.728], Fig. 3-F. Planned contrasts revealed that rats spend more time on contact with tunnels in this zone in trials T1, T2 and T3 as compared to trial H [t=-8.498, *p* < 0.001]. Then, there was a decrease in time in trials T2 and T3 in comparison to trial T1 [t = 6.558, *p* < 0.001], and the time was reduced again in trial T3 compared to T2 [t = 3.682, *p* < 0.001].

### General activity

#### Frequency of moving between the zones (left/right/transporter) of the chamber

No differences were observed in the frequency of moving between the zones of the chamber [F(3,48) = 1.095, *p* = 0.360].

### Descriptive summary of experiment #1 results

In Experiment #1, the rats were habituated to the symmetrical arrangement of the chamber with all four tunnels remaining closed. Two tunnels were opened prior to the test trials. Thus, the novelty effect took the form of opening the entrances to two of the four tunnels. The rats responded with a significant shift in their time/effort budget toward the newly opened tunnels, which was associated with a decrease in the time and effort budget allocated to a still-closed tunnel, the central zone of the chamber, and the transporter.

### Experiment #2

#### Procedure

**Fig. 4:**
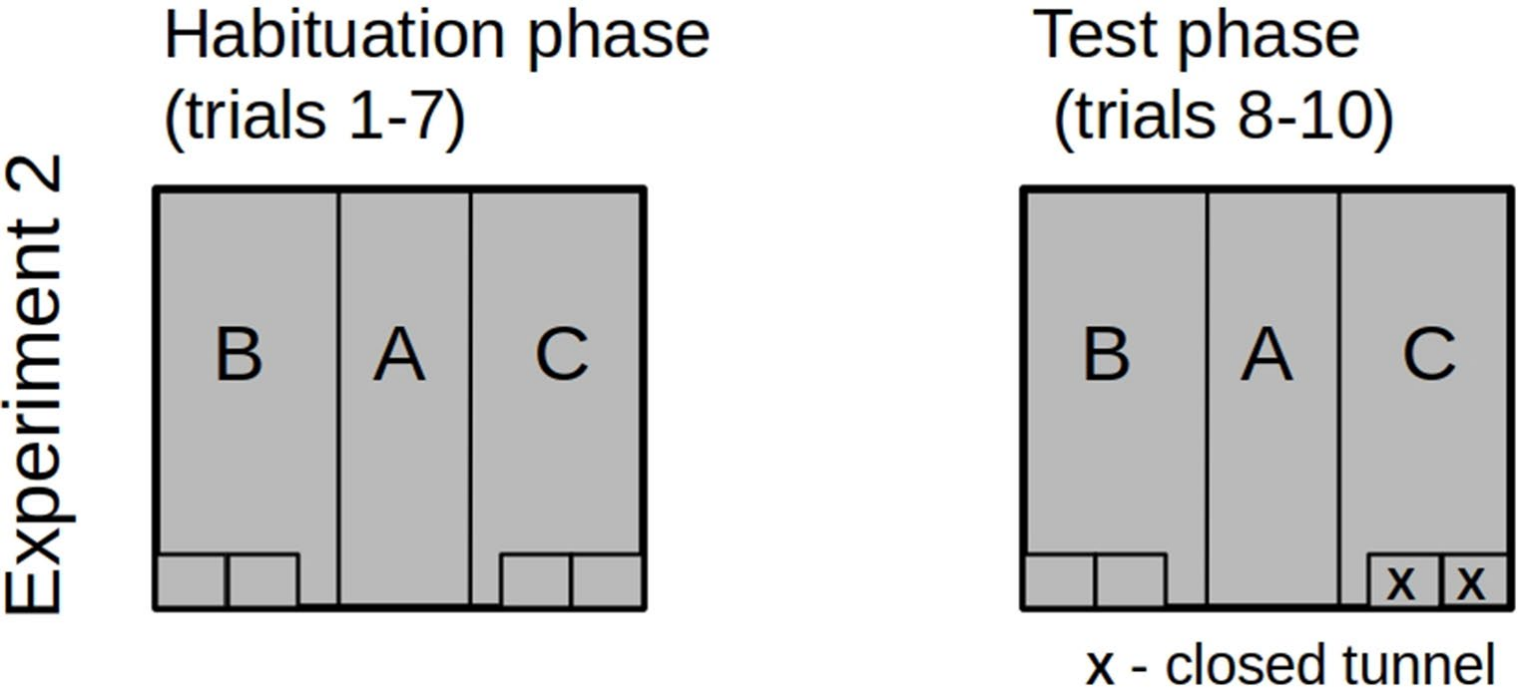
The tunnels’ configuration in the habituation and test in Experiment #2. “X” indicates a closed tunnel entrance

In experiment #2, the familiarization phase consisted of the symmetric arrangement of the chamber with all four tunnels open. Prior to the test trials, two tunnels were closed (Fig. 4). Therefore, the novelty was introduced by closing the tunnel entrances on one side of the experimental box. The change was on the left or right side of the chamber across all three trials for a given individual. To avoid the effect of lateralization or visual-auditory cues, the novelty was introduced in the right zone (as described above - Zone C) for half of the test rats and in the left zone (Zone B) for the other half (mirror image of the experimental settings).

### Results of experiment #2

#### Time budget allocation across the chamber zones

#### Time spent in the transporter

There were no significant differences across the trials [F(3,45) = 2.520, *p* = 0.07].

#### Time spent in the central zone

**Fig. 5:**
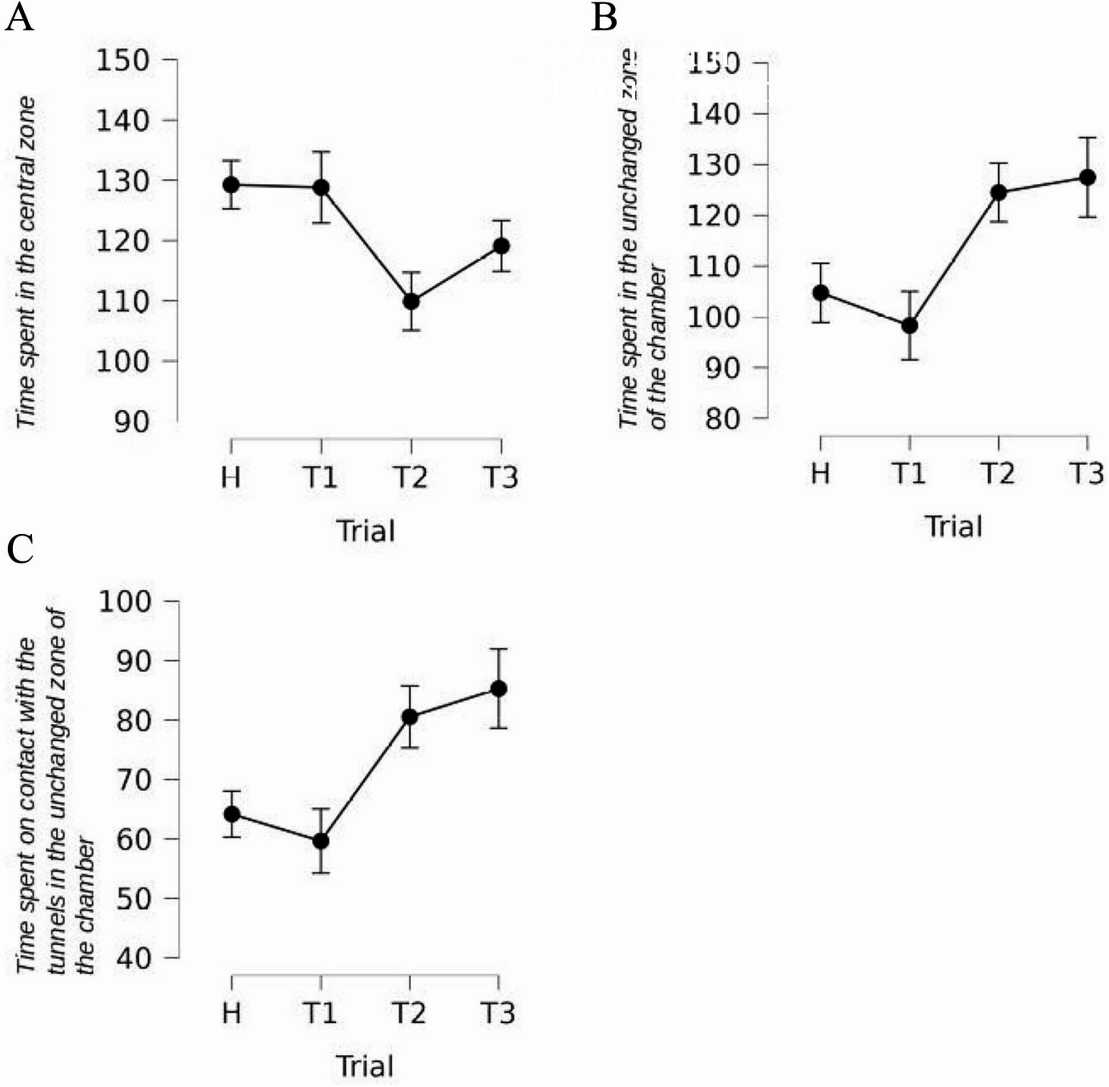
Statistically significant results of experiment #2. Time in seconds. Error bars show standard error

Animals reduced the time spent in the chamber’s central zone during the subsequent measurements [F(3,45) = 3.684, *p* = 0.019, η²*p* = 0.197]. Planned contrasts revealed a significant decline in the duration of staying in the central zone in trial T2 and T3 as compared to trial T1 [t = 2.438, *p* = 0.019] - Fig. 5-A.

#### Time spent in the unchanged zone of the chamber

Rats prolonged the duration of staying in the unmodified (therefore maintaining open tunnels) zone of the chamber [F(3,45(= 4.790, *p* = 0.006, η²*p* = 0.242]. Planned contrasts show that this change occurred in trials T2 and T3, as compared to trial T1 [t=-3.431, *p* = 0.001], Fig. 5-B.

#### Time spent in the changed zone of the chamber

There were no significant differences across the trials in the duration of staying in the modified (by closing the tunnels) zone [F(3,45) = 0.600, *p* = 0.618].

### Interactions with the open vs closed tunnels

#### Time spent on contact with the tunnels in the unchanged zone of the chamber

Rats prolonged the duration of interacting with tunnels in the unmodified (therefore maintaining open tunnels) zone of the chamber [F(3,45) = 5.337, *p* = 0.003, η²*p* = 0.262]. Planned contrasts revealed that this change occurred in trial T2 and T3, as compared to trial T1 [t=-3.534, *p* < 0.001], Fig. 5-C.

## Time spent on contact with the tunnels in the changed zone of the chamber

There were no significant differences across the trials in the duration of interacting with the tunnels in the modified (by closing the tunnels) zone [F(3,45) = 0.746, *p* = 0.530].

### General activity

#### Frequency of moving between the zones (left/right/transporter) of the chamber

There were no significant differences across the trials in the frequency of moving between the zones [F(3,45) = 1.346, *p* = 0.271].

### Descriptive summary of experiment #2 results

In Experiment #2, the rats were habituated to the symmetrical arrangement of the chamber with all four tunnels open. Prior to the test trials, two tunnels were closed. Thus, the novelty effect took the form of closing the entrances to two of the four tunnels. The rats responded by changing their time budget allocation, increasing the time spent near the continuously open tunnels. Notably, they did not reduce the amount of time spent near the newly closed tunnel. They mainly reduced the time spent in the central zone of the chamber.

### Experiment #3

**Fig. 6:**
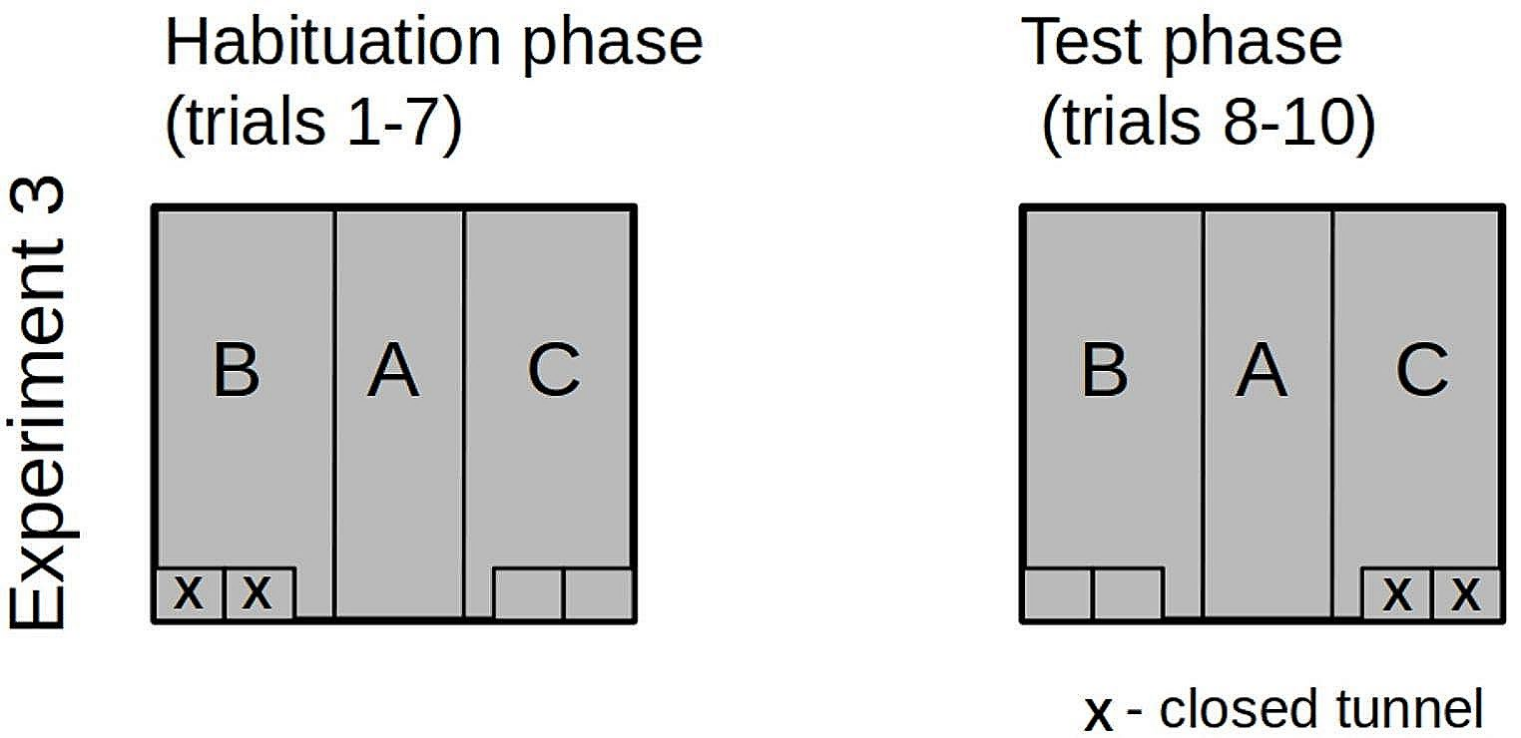
The tunnels’ configuration in the habituation and test phases in Experiment #3. “X” indicates a closed tunnel entrance

In Experiment #3, the familiarization phase consisted of the asymmetrical arrangement of the chamber, with two tunnels on one side left open and the remaining two tunnels on the other side of the chamber closed (Fig. 6). Before the test trials, the arrangement of the tunnels was changed by closing the open tunnels and opening the closed tunnels on the other side. Thus, the novelty was introduced by changing the order of the open and closed tunnels; i.e., the tunnels that were open during the familiarization trials were closed during the test trials and vice versa. The rats were randomly assigned to the correct manipulation to avoid a possible left/right preference. To avoid the effect of lateralization or visual-auditory cues, in the habituation phase, the tunnels were open in the right zone (as described above - zone C) for half of the test rats and in the left zone (zone B) for the other half (mirror image of the experimental settings).

## Results of experiment 3

### Time budget allocation across the chamber zones

#### Time spent in the transporter

No differences were observed in the duration of staying in the transporter [F(3,48) = 1.484, *p* = 0.231].

#### Time spent in the central zone

**Fig. 7:**
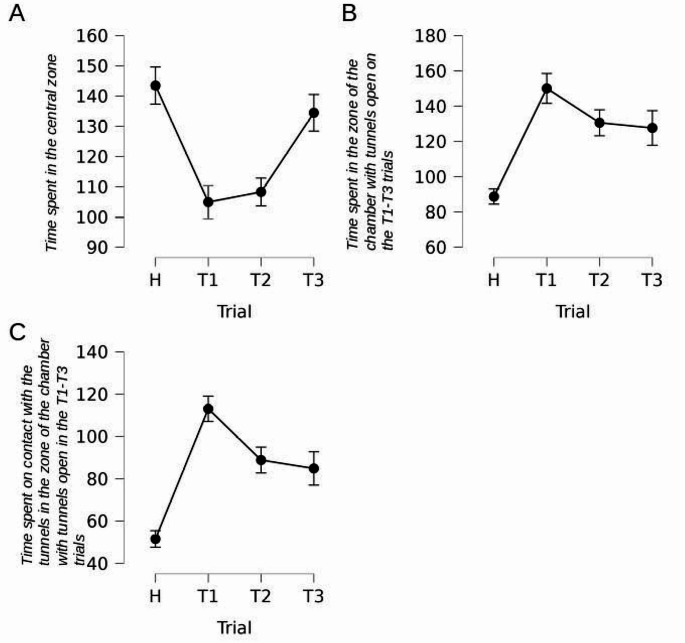
Statistically significant results of experiment #3. Time in seconds. Error bars show standard error

Rats significantly changed the duration of staying in the central zone of the chamber across the experimental trials [F(3,48) = 11.667,*p* < 0.001, η²*p* = 0.442]. Planned contrasts showed a decline in trials T1, T2, and T3, as compared to trial H [t = 4.427, *p* < 0.001]. Recovery of the duration of rats staying in the central zone of the chamber was observed in trials T2 and T3, as compared to trials T1 [t=-2.401, *p* = 0.003, with longer time in T3 compared to T2 [t=-3.312, *p* = 0.002], Fig. 7-A.

#### Time spent in the zone of the chamber with tunnels closed on the T1-T3 trials

No differences were observed in the duration of staying in the zone of the chamber with tunnels closed on the T1-T3 trials [F(3,48) = 2.479, *p* = 0.072].

#### Time spent in the zone of the chamber with tunnels open on the T1-T3 trials

Rats responded with a clear prolongation of the time spent in the chamber zone with tunnels open in the trials T1-T3 [Greenhouse-Geisser correction: F(2.135,34.164) = 10.971, *p* < 0.001, η²*p* = 0.407]. According to planned contrasts, the effect was observed in trials T1, T2, and T3 as compared to H [t=-5.289, *p* < 0.001]. However, there was a decline in the time spent in this zone in T2 and T3 compared to T1 [t = 2.206, *p* = 0.032], Fig. 7-B.

### Interactions with the open vs closed tunnels

#### Time spent on contact with the tunnels in the zone of the chamber with tunnels closed on the T1-T3 trials

No differences were observed in the duration of interacting with tunnels closed on the T1-T3 trials [F(3, 48) = 2.160, *p* = 0.105].

#### Time spent on contact with the tunnels in the zone of the chamber with tunnels open in the T1-T3 trials

Rats demonstrated a significant change in the duration of interacting with tunnels open in the T1-T3 trials [F(3,48) = 17.011, *p* < 0.001, η²*p* = 0.515]. Planned contrasts revealed a significant prolongation of the interaction with the tunnels’ time in trials T1, T2, and T3, as compared to trial H [t=-6.218, *p* < 0.001]. Then, a decline was observed in trial T2 and T3 compared to trial T1 [t = 3.487, *p* = 0.001], Fig. 7-C.

### General activity

#### Frequency of moving between the zones (left/right/transporter) of the chamber

No differences were observed in the frequency of moving between the zones [F(3, 48) = 2.492, *p* = 0.071].

### Descriptive Summary of Experiment #3 Results

In Experiment #3, the rats were habituated to the asymmetrical arrangement of the chamber, with two tunnels on one side of the chamber left open and the remaining two tunnels on the other side of the chamber closed. Prior to the test trials, the arrangement of the chamber sides was reversed. Thus, the novelty effect took the form of a change in the arrangement of the open and closed tunnels. The rats responded with a significant shift of the time/effort budget to the newly opened tunnels, which was associated with a decrease in the time and effort budget allocated to the central zone of the chamber. Notably, they did not reduce the amount of time they spent near the newly closed tunnels. Rats were confronted with two competing environmental changes: the opening of the tunnels on one side and the closure of the tunnels on the other side. Although the environmental change combined with the emergent affordance (tunnel opening) affected the rats’ behavior similarly to Experiment 1, the rats did not stop inspecting the side of the chamber with the newly closed tunnels. This is consistent with the picture obtained in Experiment #2.

## Discussion

In the present study, which consisted of three experiments, the emergent affordances at the operational level were represented by the emergent tunnel entrances, which allowed the animals to explore new sections of the test arena. The complexity of the environment was kept at a stable level throughout the experiment. Thus, animals were exposed to environmental changes in the form of emergence vs. elimination of affordances represented by open vs. closed tunnels. The general hypothesis was that under maximum control of the complexity and attractiveness of the test arena, rats would show a pronounced response to newly open tunnels (emergent affordance), while showing a lower level of exploratory activity to newly closed tunnels (disappearing affordance).

Affordances refer to the potential actions or opportunities that an environment provides for an organism (Gibson 1977; Turvey 1992; Greeno 1994; Stoffregen 2003). Like many other animals, rats have evolved to perceive and respond to these affordances in their environment. One study showed that rats shift their activity to newly opened tunnels, which represent affordances in the study, demonstrating their ability to perceive and exploit the opportunities presented by their environment (Pisula et al. 2021). However, the present study also found that rats also explore (albeit to a much lesser extent) the phenomenon of disappearing affordances (newly closed tunnels), suggesting that rats may be investing some cognitive effort to recognize when affordances in their environment are no longer available. This may be due to their evolutionary history, which has focused primarily on exploiting newly emerging affordances rather than tracking the disappearance of existing affordances (Pisula et al. 2019, 2021). Overall, the results are consistent with those of Alvernhe et al. (2012), although the significant methodological differences prevent direct comparisons. Their findings are particularly relevant in this context, as rats re-explored the closed entries. In our study, rats did not reduce the amount of time they spent near the closed tunnels in both Exp. 2 and Exp. 3. It seems that in both studies, the closure of the entrance/tunnel caused rats to make an effort to adapt to the environmental change. One might propose a concurrent hypothesis that exploring tunnels predominantly reflects shelter-seeking behavior in rats. We did not code the time spent inside the tunnels, mainly because this behavior rarely occurs. Rats visit tunnels and inspect them but do not spend time inside the tunnels. Moreover, our test arena (darkness, quietness) and the whole procedure (long habituation phase) proved to be very low-stress (Pisula et al. 2019, 2021). Therefore, shelter-seeking does not seem important in shaping rat behavior in this experimental setting.

It should be remembered that in our present study, the rats showed a clear shift in their activity toward newly opened tunnels, demonstrating their ability to perceive and take advantage of the opportunities offered by their environment. This phenomenon may be viewed as a product of natural selection that prioritizes exploiting novel resources over dwelling on the loss of existing ones. This bias towards functional novelty may have provided a selective advantage by encouraging exploration and adaptability in constantly changing environments. It should also be noted that, generally speaking, rats responded particularly eminently to a change that created a new opportunity for behavioral actions (such as hiding in the tunnel) and not to the change in the environment complexity itself. One of the interpretations of such cognitive bias towards novel affordances is that rats may experience cognitive limitations in tracking all affordances. Therefore, they would rather focus their efforts on the novel events. Their cognitive processes may be more oriented toward immediate and local sensory information rather than maintaining a detailed mental map of the environment over time. This may be related to the trade-off between the size and complexity of their brains and their ecological niche, where constant vigilance for new opportunities is critical for survival, as Heldstab et al. (2022) discussed.

In conclusion, the paradigm shift in animal research within psychology to focus on processes rather than specific species, as advocated by Vonk (2021), underscores the importance of understanding the development, function, and mechanisms of behavior across diverse biological contexts. The research presented in this paper aligns with this contemporary perspective, using rats as subjects to investigate emergent affordances and their impact on exploratory behavior. The results show a strong response in rats to recently opened tunnels, indicating a cognitive preference for exploring new opportunities (affordances) rather than focusing on vanishing ones.

The observed behavioral patterns in rats that favor the exploration of emergent affordances are consistent with the notion that evolution may have shaped a cognitive bias that prioritizes the exploitation of novel resources (Rietveld and Kiverstein 2014; Mettke-Hofmann 2017). This bias toward novelty may confer a selective advantage by promoting adaptability in changing environments. The evolutionary pressure to optimally exploit alternative foraging opportunities also shaped the behavioral mechanisms of ancestral humans, which may have resulted in a cognitive bias that prioritized the exploitation of novel resources (Park 2022). This bias may have been an adaptation to the harsh and unpredictable natural environment in which both rat and human ancestors lived. The importance of the relationship between environmental complexity and cognition evolution has also been extensively discussed by (Godfrey-Smith 2002). However, as noted by Nematipour et al. (2022), the significance of this phenomenon for understanding animal behavior may go far beyond this interpretation. We suggest that studying cognitive bias towards newly emerging affordances from a comparative psychology perspective could enhance our understanding of ecologically influenced cognitive evolution.Rats provided with an opportunity to explore tunnels may offer an ecologically valid research method.

## Data Availability

All data, protocols, recordings, and archived files will be made available upon request.

## References

[CR1] Alvernhe A, Sargolini F, Poucet B (2012) Rats build and update topological representations through exploration. Anim Cogn 15:359–368. 10.1007/s10071-011-0460-z 21915695 10.1007/s10071-011-0460-z

[CR2] Andersson A, Laikre L, Bergvall UA (2014) Two shades of boldness: novel object and anti-predator behavior reflect different personality dimensions in domestic rabbits. J Ethol 32:123–136. 10.1007/s10164-014-0401-9

[CR3] Antunes M, Biala G (2012) The novel object recognition memory: neurobiology, test procedure, and its modifications. Cogn Process 13:93–110. 10.1007/s10339-011-0430-z 22160349 10.1007/s10339-011-0430-z PMC3332351

[CR4] Arqué G, Fotaki V, Fernández D et al (2008) Impaired spatial learning strategies and Novel Object Recognition in mice Haploinsufficient for the dual specificity tyrosine-regulated Kinase-1A (Dyrk1A). PLoS ONE 3:e2575. 10.1371/journal.pone.0002575 18648535 10.1371/journal.pone.0002575 PMC2481280

[CR6] Berlyne DE (1955) The arousal and satiation of perceptual curiosity in the rat. J Comp Physiological Psychol 48:238–246. 10.1037/h0042968 10.1037/h0042968 13252149

[CR5] Berlyne DE (1963) Complexity and incongruity variables as determinants of exploratory choice and evaluative ratings. Can J Psychol 17(3):274. 10.1037/h0092883 14048839 10.1037/h0092883

[CR7] Berlyne De, Novelty and curiosity as determinants of exploratory behaviour1 (1950) Br J Psychol Gen Sect. 41:68–80. 10.1111/j.2044-8295.1950.tb00262.x

[CR8] Blaszczyk MB (2017) Boldness towards novel objects predicts predator inspection in wild vervet monkeys. Anim Behav 123:91–100. 10.1016/j.anbehav.2016.10.017

[CR9] Chrzanowska A, Modlinska K, Goncikowska K, Pisula W (2022) Rat’s response to a novelty and increased complexity of the environment resulting from the introduction of movable vs. stationary objects in the free exploration test. PLoS ONE 17:e0279006. 10.1371/journal.pone.0279006 36538520 10.1371/journal.pone.0279006 PMC9767355

[CR10] Costa VD, Tran VL, Turchi J, Averbeck BB (2014) Dopamine modulates novelty seeking behavior during decision making. Behav Neurosci 128(5):556. 10.1037/a0037128 24911320 10.1037/a0037128 PMC5861725

[CR11] Farahbakhsh ZZ, Siciliano CA (2021) Neurobiology of novelty seeking. Science 372:684–685. 10.1126/science.abi7270 33986168 10.1126/science.abi7270

[CR50] Friard O, Gamba M (2016) BORIS: a free, versatile open-source event-logging software for video/audio coding andlive observations. Methods Ecol Evol 7:1325–1330. 10.1111/2041-210X.12584

[CR12] Gibson J (1977) The theory of affordances. Perceiving, acting, and knowing: toward an ecological psychology. Erlbaum., Hillsdale, pp 67–82

[CR13] Godfrey-Smith P, Sternberg R, Kaufman J (2002) Lawrence Erlbaum, 233–249

[CR14] Greeno JG (1994) Gibson’s affordances. Psychol Rev 101:336–342. 10.1037/0033-295X.101.2.336 8022965 10.1037/0033-295x.101.2.336

[CR15] Heldstab SA, Isler K, Graber SM et al (2022) The economics of brain size evolution in vertebrates. Curr Biol 32:R697–R708. 10.1016/j.cub.2022.04.096 35728555 10.1016/j.cub.2022.04.096

[CR16] Maestripieri D (2005) On the Importance of Comparative Research for the understanding of human behavior and development: a reply to Gottlieb & Lickliter (2004). Soc Dev 14:181–186. 10.1111/j.1467-9507.2005.00296.x

[CR17] Mettke-Hofmann C (2017) Neophobia. In: Vonk J, Shackelford T (eds) Encyclopedia of animal cognition and behavior. Springer International Publishing, Cham, pp 1–8

[CR51] Modlinska K, Chrzanowska A, Pisula W (2019) The impact of changeability of enriched environment on explorationin rats. Behav Process 164:78–85. 10.1016/j.beproc.2019.04.015 10.1016/j.beproc.2019.04.015 31028795

[CR18] Modlinska K, Chrzanowska A, Pisula W (2020) Variability of enriched environment does not enhance the enrichment effect on food neophobia in rats (Rattus norvegicus). Behav Process 180:104221. 10.1016/j.beproc.2020.104221 10.1016/j.beproc.2020.104221 32835816

[CR19] Nematipour B, Bračić M, Krohs U (2022) Cognitive bias in animal behavior science: a philosophical perspective. Anim Cogn 25:975–990. 10.1007/s10071-022-01647-z 35781584 10.1007/s10071-022-01647-z PMC9334413

[CR20] Park PS (2022) The evolution of cognitive biases in human learning. J Theor Biol 541:111031. 10.1016/j.jtbi.2022.111031 35143847 10.1016/j.jtbi.2022.111031

[CR21] Pisula W (2020) Curiosity and information seeking in animal and human behavior: a review the literature and data in comparative psychology, animal cognition, ethology, ontogenesis, and elements of cognitive neuroscience as they relate to animal inquisitiveness, 2nd Edition. Brown Walker Press, Irvine

[CR23] Pisula W, Modlinska K (2020) Protocol for Measuring Free (Low-stress) exploration in rats. BIO-PROTOCOL 10: 10.21769/BioProtoc.3485 10.21769/BioProtoc.3485 PMC7842670 33654718

[CR22] Pisula W, Modlinska K (2023) Animals in search of Stimulation and Information: a review of over 10 years of our research on spontaneous exploration in rats as a response to novelty in low-stress paradigm. AB&C 10:287–303. 10.26451/abc.10.04.01.2023

[CR27] Pisula W, Turlejski K, Stryjek R et al (2012) Response to novelty in the laboratory Wistar rat, wild-captive WWCPS rat, and the gray short-tailed opossum (Monodelphis domestica). Behav Process 91:145–151. 10.1016/j.beproc.2012.06.010 10.1016/j.beproc.2012.06.010 22776746

[CR24] Pisula W, Modlinska K, Chrzanowska A (2019) Behavioural response to the environmental changes of various types in Lister-Hooded male rats. Sci Rep 9:7111. 10.1038/s41598-019-42924-1 31068618 10.1038/s41598-019-42924-1 PMC6506482

[CR25] Pisula W, Modlinska K, Chrzanowska A, Goncikowska K (2021) Response to novelty induced by change in size and complexity of familiar objects in Lister-Hooded rats, a follow-up of 2019 study. Sci Rep 11:10281. 10.1038/s41598-021-89289-y 33986341 10.1038/s41598-021-89289-y PMC8119972

[CR26] Pisula W, Modlinska K, Goncikowska K, Chrzanowska A (2022) Decrease in the rewarding value of spatial novelty due to the contamination of the stimulus field with light – evidence from a free exploration test involving rats. Behav Process 202:104738. 10.1016/j.beproc.2022.104738 10.1016/j.beproc.2022.104738 36064066

[CR28] Rietveld E, Kiverstein J (2014) A Rich Landscape of Affordances. Ecol Psychol 26:325–352. 10.1080/10407413.2014.958035

[CR29] Stoffregen TA (2003) Affordances as properties of the animal–environment system. Ecol Psychol 15:115–134

[CR30] Takola E, Krause ET, Müller C, Schielzeth H (2021) Novelty at second glance: a critical appraisal of the novel object paradigm based on meta-analysis. Anim Behav 180:123–142. 10.1016/j.anbehav.2021.07.018

[CR31] Turvey MT (1992) Affordances and prospective control: an outline of the Ontology. Ecol Psychol 4:173–187. 10.1207/s15326969eco0403_3

[CR32] Vergara-Ovalle F, Ayala-Guerrero F, Rosas C, Sánchez-Castillo H (2023) Novel object recognition in Octopus maya. Anim Cogn 26:1065–1072. 10.1007/s10071-023-01753-6 36809584 10.1007/s10071-023-01753-6 PMC10066149

[CR33] Vonk J (2021) The journey in comparative psychology matters more than the destination. J Comp Psychol 135:156–167. 10.1037/com0000279 34180708 10.1037/com0000279

[CR34] Wagman JB, Langley MD, Farmer-Dougan V (2017) Doggone affordances: canine perception of affordances for reaching. Psychon Bull Rev 24:1097–1103. 10.3758/s13423-016-1183-6 28168679 10.3758/s13423-016-1183-6

